# The Impact of Conflict on Immunisation Coverage in 16 Countries

**DOI:** 10.15171/ijhpm.2018.127

**Published:** 2018-12-30

**Authors:** John Grundy, Beverley-Ann Biggs

**Affiliations:** ^1^College of Public Health, Medical and Veterinary Services, Cairns Campus, James Cook University, Douglas, QLD, Australia.; ^2^Department of Medicine, Doherty Institute, University of Melbourne, Melbourne, VIC, Australia.; ^3^Victorian Infectious Diseases Service, Royal Melbourne Hospital, Parkville, VIC, Australia.

**Keywords:** Immunisation, Conflict, Displaced Populations, Refugees, Equity, GAVI

## Abstract

**Background:** Military conflict has been an ongoing determinant of inequitable immunisation coverage in many low- and middle-income countries, yet the impact of conflict on the attainment of global health goals has not been fully addressed. This review will describe and analyse the association between conflict, immunisation coverage and vaccine-preventable disease (VPD) outbreaks, along with country specific strategies to mitigate the impact in 16 countries.

**Methods:** We cross-matched immunisation coverage and VPD data in 2014 for displaced and refugee populations. Data on refugee or displaced persons was sourced from the United Nations High Commissioner for Refugees (UNHCR) database, and immunisation coverage and disease incidence data from World Health Organization (WHO) databases. Demographic and Health Survey (DHS) databases provided additional data on national and sub-national coverage. The 16 countries were selected because they had the largest numbers of registered UNHCR "persons of interest" and received new vaccine support from Global Alliance for Vaccine and Immunisation (GAVI), the Vaccine Alliance. We used national planning and reporting documentation including immunisation multiyear plans, health system strengthening strategies and GAVI annual progress reports (APRs) to assess the impact of conflict on immunisation access and coverage rates, and reviewed strategies developed to address immunisation program shortfalls in conflict settings. We also searched the peer-reviewed literature for evidence that linked immunisation coverage and VPD outbreaks with evidence of conflict.

**Results:** We found that these 16 countries, representing just 12% of the global population, were responsible for 67% of global polio cases and 39% of global measles cases between 2010 and 2015. Fourteen out of the 16 countries were below the global average of 85% coverage for diphtheria, pertussis, and tetanus (DPT3) in 2014. We present data from countries where the onset of conflict has been associated with sudden drops in national and sub-national immunisation coverage. Tense security conditions, along with damaged health infrastructure and depleted human resources have contributed to infrequent outreach services, and delays in new vaccine introductions and immunisation campaigns. These factors have in turn contributed to pockets of low coverage and disease outbreaks in sub-national areas affected by conflict. Despite these impacts, there was limited reference to the health needs of conflict affected populations in immunisation planning and reporting documents in all 16 countries. Development partner investments were heavily skewed towards vaccine provision and working with partner governments, with comparatively low levels of health systems support or civil partnerships.

**Conclusion:** Global and national policy and planning focus is required on the service delivery needs of conflict affected populations, with increased investment in health system support and civil partnerships, if persistent immunisation inequities in conflict affected areas are to be addressed.

## Background

Achieving equity in immunisation outcomes has received increased focus from global health agencies, as they expand efforts to control, eliminate and eradicate vaccine-preventable diseases (VPDs). Geographic location, gender, and socio-economic status are important factors affecting equitable access to immunisation services.^1^ As well, military conflict is a major contextual determinant of lower immunisation coverage and is ongoing in many developing countries. For example, fighting between government military and insurgents in Pakistan has resulted in the displacement of millions, with the resulting spread of wild poliovirus to other parts of the country.^2^ In Afghanistan, lack of security and immunisation coverage were negatively associated, regardless of availability of resources.^3^ More recently in the Ukraine, an outbreak of fighting resulted in a collapse in vaccination coverage and outbreaks of both polio and measles.^4^ Such studies are important in understanding the effect of conflict on immunisation within countries, as well as on strategies to mitigate the impacts of conflict. However, the impact of conflict on the attainment of global health goals has not been fully addressed. In this review, we describe and analyse the impact of conflict on immunisation programs across 16 high-risk countries that received support from Global Alliance for Vaccine and Immunisation (GAVI), the Vaccine Alliance, with a view to generating themes that inform global and national policy on reducing immunisation inequities in conflict-affected settings.

## Methods and Sources of Data

Sources of data for this review include data bases of the World Health Organization (WHO) (reporting on vaccine coverage^5^ and disease incidence^6^), Demographic and Health Survey (DHS) data, and country planning and reporting documents available through the GAVI Country Hub.^7^ The DHS data, in combination with disease outbreak and emergency response situation reports published online through United Nations sources, provided information on sub-national immunisation coverage and VPD outbreaks.

To provide background contextual information on the issue of immunisation and conflict, a search was conducted through the PubMed database^8^ using the title search terms ‘immunisation’ and ‘conflict,’ (3 responses) ‘immunization’ and ‘conflict,’ (5 responses), ‘vaccination’ and ‘conflict’ (4 responses), ‘immunisation’ and ‘war’ (1 response) and ‘immunization’ and ‘war’ (6 responses) and finally, ‘vaccination’ and ‘war’ (23 responses). Of these studies, none undertook a global review of the issue of conflict and immunisation, although one study attempted to draw global lessons from the experience of immunisation and conflict in Nigeria, Pakistan, and Somalia.^9^ Other studies from Nigeria,^10^ Sierra Leone,^11^ Central African Republic (CAR),^12^ Syria,^13^ and Nepal^14^ were highly country specific, and review how conflict effects service delivery strategies such as campaigns and routine immunisation in conflict affected areas within national borders.

In this review, we adopt a wider-angle view of the impact of conflict on immunisation. We do this by describing and analysing 3 sets of immunisation planning and reporting documents from 16 countries. These documents include national multiyear immunisation plans, health system strengthening (HSS) strategies and GAVI annual progress reports (APRs).^7^ These planning and reporting documents were accessed through the GAVI Country Hub website (https://www.gavi.org/country/). This site provides information on levels of vaccine and financing for each country that is eligible for GAVI support. The site also provides access to a set of country documents, including the above mentioned national multi-year plans, APRs (prepared by each country) and HSS proposals financed through GAVI and national governments. These 3 sets of documents were emphasized in this analysis as they provide a consistent approach as to how countries report internationally on immunisation.

We applied search terms in these documents using the United Nations High Commissioner for Refugees (UNHCR) classifications (refugees, displaced populations, internally displaced person [IDP] or ‘displaced’ populations, stateless persons, returnees) to detect specific responses by national planners for the provision of immunisation services to vulnerable or conflict-affected populations. These same search terms were applied for the APR, national comprehensive multi-year plans for immunisation (cMYP) and for a country’s HSS strategy. Frequencies of mention of search terms were presented in tabular format in an Excel spreadsheet. Data were also recorded here on documented impacts of conflict on immunisation including documentation of immunisation strategy in conflict-affected areas. The data from the above sources was then cross referenced with UNHCR data for 2014 as well as with immunisation coverage (2014) and disease reporting data (WHO 2010–2015).

Countries were selected for review based on 2 criteria; firstly, that the country had 500 000 or more “persons of interest” as classified by UNHCR, and secondly that the country was eligible for international Global Health Initiative support through GAVI.

### Analysis and Limitations of Study

The review presents an overview of the available information on conflict-affected populations in the 16 review countries, and data on immunisation coverage and VPD outbreaks. Wherever possible, coverage and outbreak data are tracked back to sub-national regions where conflict is reported. We examine the availability and accessibility of services in conflict areas, with a focus on human resources, infrastructure, and service delivery. We also reviewed national planning and reporting documents to detect country strategies that respond to service gaps in conflict-affected areas.

An important methodological limitation of this paper is that other social and geographic variables, such as illiteracy, poverty and remoteness may confound the impact of conflict on immunisation access. We have tried to minimise this risk by adopting a wide multinational comparison of the impact of conflict on immunisation access and coverage and disease outbreaks, and wherever possible analysing subnational data and linking this data to reporting of conflict in the same sub-national areas.

We have also utilised peer-reviewed sources and evidence from within country case studies to confirm overlap of conflict settings with low coverage. We cite national countries own reporting documents to confirm the direct linkages between health system collapse, insecurity, and low coverage in sub-national regions of a given country. Based on these 3 sources, we determine the association between conflict conditions and a country’s immunisation service accessibility, coverage, and reporting of disease outbreaks. The peer reviewed articles were used to provide background information on the statement of immunisation and conflict as a global health problem. In contrast, the review of the planning and reporting documentation was used to systematically describe and analyse the policy and planning stance of national governments towards the issue of immunisation in conflict settings. The main rationale for selection of GAVI eligible countries was to enable a systematic cross-country comparison of immunisation strategy in conflict settings utilizing common data sources and reporting formats.

## Results

### Data on Conflict-Affected Populations in 16 Review Countries

**Figure 1:**
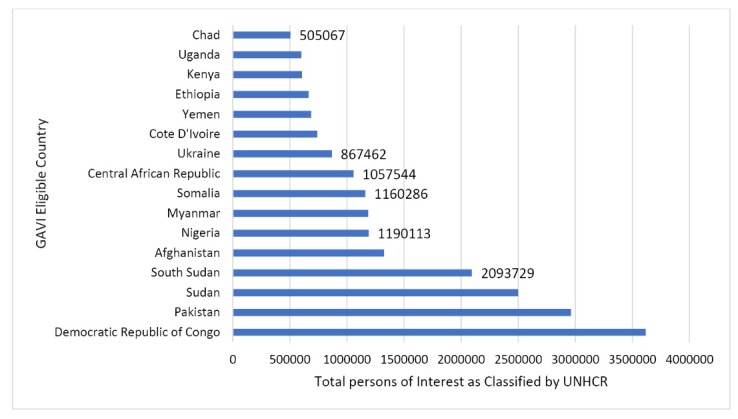
Persons of Interest as Classified by the UNHCR in 2014 in 16 Conflict-Affected Countries.^15^

Note: Countries with data labels have coverage below 50% DPT3 WHO UNICEF Estimates 2014. See Figure 2. Abbreviation: UNHCR, United Nations High Commissioner for Refugees.

The 16 countries have over 21 million persons of interest as categorised by UNHCR, of whom 60% are IDPs, 22% refugees and 18% other UNHCR category. Persons of interest in this sample ranges from 3.6 million in the Democratic Republic of Congo (DRC) to just over 500 000 in Chad (Figure 1).

These figures underestimate the true number of people affected by conflict, as many in conflict areas do not become displaced. However, by focussing on displaced persons and refugees (including other ‘persons of interest’ categories such as ‘stateless populations’), it is possible to track policy and planning responses for arguably the most disadvantaged and vulnerable populations affected by conflict.

### Immunisation Coverage in Conflict-Affected Countries

**Figure 2:**
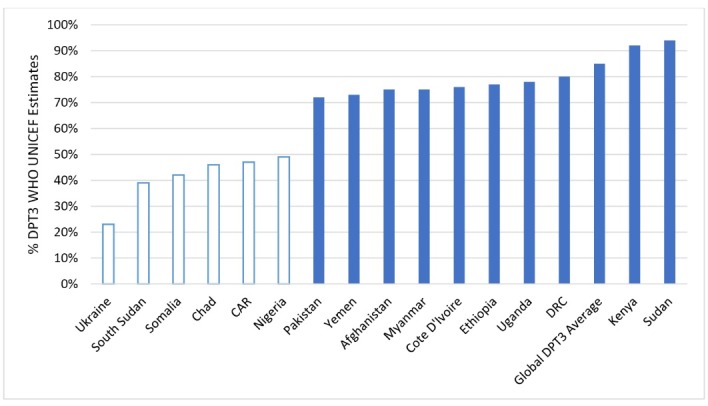
DPT3 Immunisation Coverage 2014 (WHO and UNICEF Estimates) in 16 GAVI Eligible Conflict-Affected Countries, With Clear Bars Showing Countries With Coverage <50% Coverage.^5^

Abbreviations: DPT3, diphtheria, pertussis, and tetanus; WHO, World Health Organization; UNICEF, United Nations Children’s Fund.

WHO data (2014) show that 14 of 16 conflict-affected countries have immunisation coverage below the global diphtheria, pertussis, and tetanus (DPT3) average of 85% (based on WHO and United Nations Children’s Fund [UNICEF] published estimates). Six of 16 countries have DTP3 coverage below 50% (see Figure 2).

In total there were 6 874 201 UNHCR classified persons of interest in the 6 countries in 2014 with DPT3 coverage below 50% (WHO UNICEF Estimates) (see Figures 1 and 2). Surveys and peer-reviewed sources indicate that these lower national rates are driven by pockets of very low coverage in subnational regions, many of which are affected by current or historical conflict.

**Figure 3:**
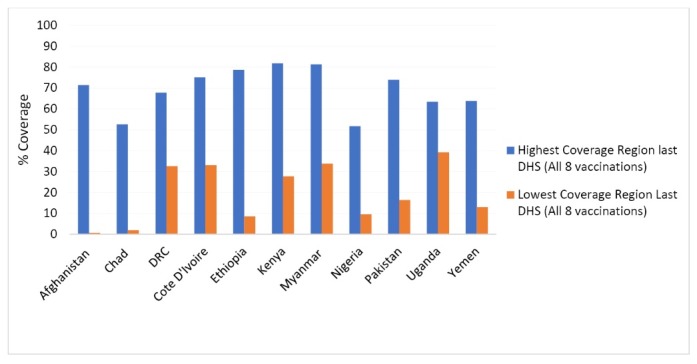
Coverage of 8 Vaccinations for the Highest and Lowest Coverage Regions Within Countries.^16^Abbreviations: DHS, Demographic and Health

Figure 3 illustrates the gap between regions within countries with the highest and lowest vaccination coverage rates (of all 8 basic vaccinations of BCG, DPT1-3, Polio 1-3 and measles vaccinations) in the 11 countries with available data in the last reported DHS survey (2011 and 2015).

Notwithstanding the situation of the very limited access by the poor to immunisation services in urban areas where displaced persons migrate to from rural areas affected by conflict, aggregated population surveys still show that the highest coverage rates are often concentrated in central urban areas such as Kinshasa in DRC, Addis Adeba in Ethiopia, Mandalay in Myanmar, Islamabad in Pakistan, Kampala in Uganda, and Aden in Yemen. In contrast, lower coverage rates are associated with remote areas and conflict. In DRC, the lowest coverage is in Equateur province, which has been the location of ongoing conflict between the police and armed militants, with Médecins Sans Frontieres (MSF) reporting up to 50 000 displaced persons in 2010.^17^ In Pakistan, the lowest regional coverage is in Balochistan (16% fully immunised),^18^ where there has been ongoing insecurity for many years. In Nigeria, very low coverage is experienced in the North West, also an area of ongoing civil unrest. In Myanmar, the second lowest regional coverage is in Rakhine State, where inter communal violence has occurred over the last 3 to 4 years. In Kenya, the lowest coverage is in Mandera, a region bordering Somalia with recent cross border attacks by Islamist group al-Shabbab.^19^ This conflict has been ongoing since 2011. Finally, in Yemen, the lowest regional coverage has been in Sadah, a remote north-western province of the country heavily implicated in the origins and persistence of the current civil war.^20^

As conflicts have been going on for decades in such countries as Myanmar, Pakistan and Afghanistan, and since conflicts are often contained in smaller sub regions, it is often difficult to provide a before and after conflict picture of national immunisation coverage. However there have been some recent cases that illustrate how national conflicts can lead to a collapse in healthcare and immunisation services access.

Between 2012 and 2013 in Ukraine, immunisation coverage had been maintained at 76% (DPT3) (WHO UNICEF Estimates) after which it declined sharply to 23% in 2014 following the commencement of conflict.^21^ It declined further to 19% in 2016. In Yemen between 2013 and 2014, the country had coverage levels of 71% and 73% respectively (DPT3 WHO UNICEF Estimates), after which the coverage declined to 47% in 2015 following the onset of conflict.^22^ At independence in 2011, the DPT3 coverage in South Sudan was 75%. But following commencement of civil conflicts post-independence, the coverage declined sharply to 46% in 2014 (DPT3 WHO UNICEF Estimates).^23^

### Vaccine Preventable Disease Outbreaks in Conflict-Affected Countries

Not surprisingly, pockets of low coverage in conflict-affected areas contribute to VPDe outbreaks (Table 1). This is most marked in the case of polio where the total number of reported cases between 2010 and 2015 in the 16 review countries (2255 cases) represented 67% of all global cases (3357 cases), even though these countries represent just 12% of the global population.

**Table 1:** Global Polio and Measles Incidence (2010-2015) and Incidence in the 16 Countries^6,24^

Total Global Population 2015	7 346 705 000
Total population in the 16 countries in 2015	891 277 274
% Global population in the 16 countries in 2015	12.1%
Total global polio cases 2010-2015	3357
Total polio cases in the 16 countries 2010-2015	2255
% Polio cases in the 16 countries as a percentage of all global cases (2010-2015)	67%
Total global measles cases 2010-2015	1 688 098
Total measles cases in the 16 countries	663 497
% Measles cases in 16 countries 2010-2015 as a percentage of all global cases (2010-2015)	39%

Table 2 describes disease outbreaks reported by subnational regions affected by conflict. A common theme in the analysis of these cases is the association of disease outbreaks with population displacement. Overall, since 2010, 13 of the 16 countries have experienced ether wild polio or circulating vaccine derived polio outbreaks since 2010.

**Table 2:** Polio Outbreaks in the 16 Countries

**Country**	**Reported Wild Polio Virus and Circulating Vaccine Derived Polio Virus**
Afghanistan	Polio cases in 2016 were concentrated in the southern regions and along the border with Pakistan. Four cases were reported in 2016 from Bermel District bordering Pakistan, which is controlled by antigovernment forces.^26^ The total number of WPV1 cases reported in Afghanistan in 2018 is 12 (reported as of September 2018).^26^
CAR	Although no indigenous cases have been reported since 2000, imported cases were reported from Southern Chad and DRC.^27^
Chad	An outbreak was reported in 2010 from a newly imported case from Northern Nigeria, with absent/destroyed health infrastructure across Chad facilitating further spread.^28^ Chad experienced outbreaks of both wild poliovirus type 1 (WPV1 – 65 cases in 2011) and wild poliovirus type 3 (WPV3 – 3 cases) in 2011.^29^
Cote D’Ivoire	17 cases of polio re-emerged in the north of the country following the crisis in the north.^30^ Since 2002 the country has been divided between the rebel-held north and the government-controlled south, with many healthcare workers fleeing from the north.^31^ In 2011 Côte d'Ivoire experienced an outbreak of WPV3 with 3 new cases reported with onset of paralysis in 2011.^32^
DRC	In 2010, an estimated 2.6 million people lived away from home in DRC. There were 100 polio cases in 2010, and in 93 cases in 2011. Efforts to prevent polio have been complicated by conflict and insecurity including displacement of 2.5 million people.^33^ Two separate cVDPV2s have been confirmed in 2017.^34^
Ethiopia	Highest risk areas for polio transmission were from the Somali region of Ethiopia, where the last cases occurred in 2013.^35^ There were 10 confirmed WPV cases in 2013. The affected area is characterised by insecurity, weak infrastructure and communication.^36^ The country is currently (2018) conducting a polio campaign to focus on pastoralist, refugees, IDPs, hard to reach and border areas and cross-border surveillance with Somalia.^37^
Kenya	A 4-month-old girl from near Dadaab (refugee camp with estimated population of 500 000) developed ADP in 2013. Two contacts tested positive for WPV1. Risk is considered high in this region, due to large scale population movements across the Horn of Africa.^38^
Myanmar	Two cases of cVDPV were detected in Rakhine State in 2017, the location of recent communal conflicts, and where in some of the Townships only 27% of children received 3 doses of polio vaccine.^39^
Pakistan	54 polio cases reported in 2015, with cases concentrated in conflict affected border regions.^40^ In 2016, cVDPV2 was detected from environmental samples in Quetta, Balochistan. 19 WPV cases were also detected in the same year. The area affected is part of a cross border common reservoir for WPV1 that extends into Southern Afghanistan.^41^
Nigeria	Two polio cases were detected in Northern province of Borno, demonstrating the need to prioritise services in the Lake Chad Region (often affected by conflict and large population movements).^42^ The Government reported 3 laboratory-confirmed WPV1 cases in 2016 from Borno State.^43^
Somalia	During a polio outbreak in 2007, there were 228 cases, mostly from IDP.^44^ Circulation of cVDPV2 has been confirmed in Somalia in 2018.^45^
South Sudan	2 cases of cVDPV were confirmed from IDPs in Unity State (conflict-affected region of the South) in 2014.^46^
Sudan	The last reported polio cases were in 2009, and the country has been polio free since then. High risk areas remain in border areas with Chad in Darfur State.^47^ The earlier outbreaks were initially restricted to southern Sudan and western Ethiopia, after which cases spread to northern Sudan (in Khartoum and Port Sudan).^48^
Uganda	A polio case detected in the Bugiri district in mid-October 2010.^49^
Ukraine	Low coverage is amplified by ongoing conflict and displacement in the east. Two cases of circulating vaccine-derived poliovirus type 1 have been confirmed in 2015,^50^ along with 3667 cases of rubella, 2937 cases of pertussis, and 995 cases of mumps.^4^
Yemen	There are an estimated 14.8 million that have no access to health services, with WHO launching large scale campaigns to prevent polio and measles.^51^

Abbreviations: WPV1, wild polio virus type 1; DRC, the Democratic Republic of Congo; cVDPV2s, circulating vaccine-derived poliovirus type 2s; IDP, internally displaced person; ADP, acute flaccid paralysis; WHO, World Health Organization; WPV, wild polio virus; WPV 3, wild polio virus type 3; cVDPV, circulating vaccine-derived poliovirus.

**Figure 4:**
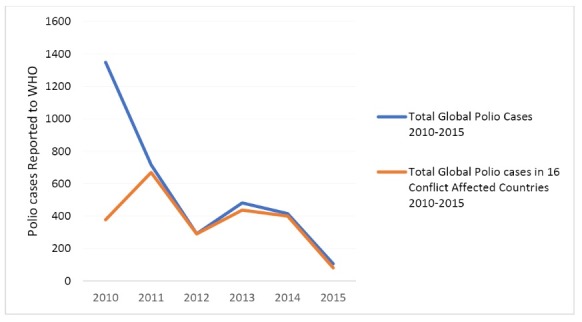
Trends in Reported Polio Cases Globally and in the 16 Countries.^6^ Abbreviation: WHO, World Health Organization.

As illustrated in Figure 4, although polio cases have been tracking down over the last 5 years in line with the trend globally, 76% of polio cases were concentrated in the countries examined in this review in 2015, with evidence that many cases originated within conflict-affected zones or in displaced populations within these countries.

These findings are consistent with a recently published global review of polio eradication activities, which concluded that the incidence of polio was high in areas with increased conflict and instability, and that conflict resulted in the re-emergence of polio in otherwise polio-free countries.^52^

### Documented Impact of Conflict on Immunisation Services

#### Health Services

The impact of conflict on immunisation services is widely reported in country planning documents, and focus mostly on reduced accessibility of services, lower coverage, and increased outbreak risk, destroyed health infrastructure and vaccine logistics, and depleted human resources. In Sudan, war and theft are reported as continuing threats to vaccine and logistics systems,^53^ whereas in South Sudan there is an absence of health facilities in conflict- affected areas altogether, with only 44% of the population living within 5 km of a health facility.^54^ Ongoing conflicts in rural areas of Afghanistan create security problems with shipment of equipment to provinces. Insecurity is continuously threatening cold chain, transportation, and storage of vaccines.^55^

In Pakistan, there is a lack of specific service provision for IDPs, who instead rely on routine services that are inadequate for the existing population.^56^ In South Sudan, an estimated one million people have fled across borders, which has led to lack of clarity on population denominators. There is delayed implementation due to difficult or no access in some counties in South Sudan because of the armed conflict (particularly in the 3 most affected states of Jonglei, Unity, and Upper Nile) resulting in delayed vaccine introductions.^57^ Most facilities are not conducting outreach in these locations.^58^ In Afghanistan, an estimated 2.5 million live in insecure regions, mostly in the south, southeast, and west.^59^ There is much lower access to immunisation campaigns in these regions, with reduced movements of beneficiaries due to insecurity. In Yemen, both measles rubella (MR) and inactivated polio vaccine (IPV) vaccine introduction were postponed until 2015 due to security unrest.^60^

Planning for displaced populations is very limited. Only one of 14 multiyear plans (14 out of 16 were available) contained the search terms “IDP” or “displaced population.” Despite UNHCR reporting over 600 000 refugees and asylum seekers in Kenya in 2014, neither the multiyear plan for immunisation or APR to GAVI (2014) makes any mention of services for these groups.^61^ This was also the case for the over 800 000 stateless persons in Myanmar. Other than mentioning low coverage in Rakhine State, there are no specific strategies to expand services for this vulnerable group.^62^ Nigeria makes no mention of strategies to expand access to immunisation services for the over 1.1 million UNHCR persons of interest in its multi-year immunisation plan.^63^

These findings are consistent with an independent review of applications for GAVI funding from 19 countries in 2014 (of which 8 were identified by reviewers as being in conflict). It was found that “populations affected by conflict are either not mentioned in proposals (invisible), or strategies are not described as to how these populations will be reached.”^64^

#### Impact on Human Resources

A common theme in national documentation is the impact of conflict on human resources availability, retention, competency, and distribution. In some of these cases, gaps in human resources are attributable to health workers fleeing conflict-affected areas. In Cote D’Ivoire, the country has been divided between the rebel-held north and the government-controlled south. Many healthcare workers have fled the north, with UNICEF reporting serious consequences for children.^65^ In some cases, health workers have been subject to acts of violence. In Pakistan, there was a targeted killing of polio workers resulting in the death of 22 polio workers and 4 police officers,^66^ and in Nigeria, at least 9 young women working on a polio vaccination campaign were targeted and killed by gunmen in 2 separate incidents in Kano, the regional capital of Northern Nigeria in 2013.^67^

There is a critical shortage of health workers in South Sudan, with a midwife/nurse to population ratio of 0.2 per 10 000.^54^ In Somalia, deterioration of the security situation in 2012 meant that the recruitment of Lady Health Workers was hampered in South Central zones.^68^ In North Sudan, mechanisms for retention and equitable deployment in remote and conflict-affected areas are not well developed, resulting in the location of over 70% of the health workforce in urban areas.^69^ In Myanmar, recruitment and retention of the health workforce in conflict-affected States bordering China and India has been a long-standing program challenge, necessitating the implementation of “special outreach” programs to reach underserved populations.^70^ Meanwhile, in the Ukraine, one WHO report found that only 30% of the medical personnel were left to care for the sick in one city in the conflict-affected east of the country. In Donetsk Oblast there are 10%-15% fewer medical personnel compared to the 85 000 that were based there before the crisis, leading to a collapse in polio immunisation coverage to between 30% and 40%.^71^ The human resource depletion in conflict-affected areas in some cases is leading to a strengthened role for non-governmental organisations (NGOs) in settings such as CAR,^72^ Afghanistan,^73^ and in Yemen.^74^

#### Impact on Information and Planning

The limited mention of displaced people in planning and HSS strategies raises real concern that such populations are not being adequately included in population denominators, or in consultation or decision-making processes. For example, South Sudan reports that over one million people have migrated from the 3 most affected States to other countries, and leaves open the question of the destination of these people and the geographic denominator to which they will now belong.^58^ Over 500 000 displaced persons are in camps in Kenya, but there is lack of mention of this group in the APRs.^61^ In Myanmar, years of internal conflict along border regions with China have led to migratory movements to harder to reach areas, with primary care midwives not including such populations in head counts due to barriers to access.^70^

### Country Strategies to Expand Access of Immunisation Services to Populations Affected by Conflict

Most countries describe a mix of strategies for service delivery in conflict-affected areas including campaign and outreach services, supported by civil society partnerships and volunteer networks that are tailored to local security conditions (see Table 3).

**Table 3:** Service Delivery Strategies for Conflict-Affected Populations Described in APRs (2014), HSS Proposals or the Most Recent Multi-Year Immunisation Plan

**Country**	**Service Delivery Strategy Identified in National Policy and Planning Documentation**
Afghanistan	Increase immunisation delivery points, aggressive mobile outreach to isolated communities, and provision of services through private providers in insecure areas. This includes contracting out of health service delivery to NGOs in 31 out of 34 provinces. Locally tailored solutions through provincial management structures in insecure areas are considered important.
Chad	Implementation of SIAs are conducted to address gaps in routine services. There is a high reliance on NGOs and international support to provide services for refugees in the east and south of the country.
CAR	Advocacy measures are conducted with multinational forces for improved security to support vaccine introductions. There are activities to support intensification of routine immunisation, and there are increased roles for NGOs in zones of insecurity.
Cote D'Ivoire	Despite reports of over 700 000 stateless persons, as well as instability in the north and east of the country, no specific strategies are described for conflict-affected populations.
DRC	A plan for refugees is described, including an emergency stock of vaccines and development of guidelines for immunisation for displaced populations. Elsewhere it states that specific strategies to reach the children in health zones with armed conflicts are not yet developed.
Ethiopia	Fixed immunisation posts have been established at cross border sites where there are large population movements.
Kenya	No specific strategy is described for displaced populations, despite this country having the largest displaced population camp in the world. The comprehensive multiyear plan identifies a high reliance on NGOs, which operate 54% of health facilities in the country.
Myanmar	Special outreach programs are implemented in remote areas and those affected by armed conflict. Planning documents identify increased roles for NGOs in border areas or areas under non-government control.
Nigeria	No specific strategies are described in 3 documents (APR, HSS, or cMYP).
Pakistan	The country uses existing services to reach displaced populations. Vaccinators utilise 60-80 days per year for national Immunisation Days strategy.
Somalia	Child Health Days are implemented in all urban, rural, and hard to reach areas, although security was viewed as a barrier to implementation. The multi-year plan for immunisation also mentions expanding routine immunisation outlets for IDPs.
The Sudan	Plans are described to (*a*) implement integrated service delivery strategy in emergency settings, elsewhere described as "accelerated routine activities." (*b*) Expand immunisation coverage in security compromised areas largely through limited "hit- and- run or acceleration campaign approach" facilitated by CSOs and NGOs. (*c*) open a channel of communication with armed groups through local leaders and UN agencies along with involvement of NGOs.
South Sudan	No specific strategies are described in 2 available national documents (GAVI, APR, and HSS). However, the HSS strategy does outline contractual/MOU mechanisms with civil society organisations. Elsewhere UNICEF and the World Food program have initiated a RRM using food distribution and support registration and health services for conflict affected populations.^75^
Uganda	No specific strategies are described, despite barriers to immunisation being noted in the conflict-affected north.
Ukraine	No data available.
Yemen	Enhancement of routine immunisation in conflict-affected areas is identified, including more frequent outreach and mobile strategies. Increased roles for CSOs in conflict areas that lack public services are also described.

Abbreviations: NGOs, non-governmental organisations; SIAs, supplementary immunisation activities; APR, annual progress report; HSS, health system strengthening; cMYP, comprehensive multi-year plans for immunisation; IDPs, internally displaced people; CSOs, civil society organizations; DRC, the Democratic Republic of Congo; UNICEF, United Nations Children’s Fund; RRM, rapid response mechanism; MOU, memoranda of understanding; UN, United Nations; CAR, Central African Republic.

Eight of 16 countries do not describe a specific strategy for conflict-affected situations in any of the 3 planning documents. In the multiyear plans for immunisation, only 1 out of the 16 countries mention the term “IDP” or “displaced” populations in their national strategic planning document, reflecting an overall lack of planning emphasis on the needs of these groups.

Seven countries describe an enhanced role of NGOs/civil society organizations (CSOs) in conflict-affected settings with services contracted out to NGOs in 31 of the 34 provinces of Afghanistan.^55^ Planners observed in Myanmar that in a “complex landscape of remote, geographically dispersed, non-government control and border areas together with migrant and other ethnic groups, increasing access to immunisation is only successful through the involvement of CSOs and NGOs.”^70^

Six countries describe non-routine campaign or additional outreach services as main strategies, reflecting both the dearth of permanent health workforce and infrastructure in conflict-affected regions. Only 2 countries describe a specific communication strategy with combatants or local authorities to enable access to populations in conflict-affected areas. In the DRC, the annual report to GAVI in 2014 states that “specific strategies to reach the children in remote areas were not established, particularly in health zones with armed conflicts and having no help.”^76^ In the Annual Reports to GAVI, only 3 out of 14 countries mention “IDP” or “displaced” populations, despite the fact UNHCR reports significant numbers of these populations in these countries (ranging from 0.7% of the population in Ethiopia to 21.6% of the population in CAR).

### Global Health Initiative Financing in Conflict-Affected States

**Figure 5:**
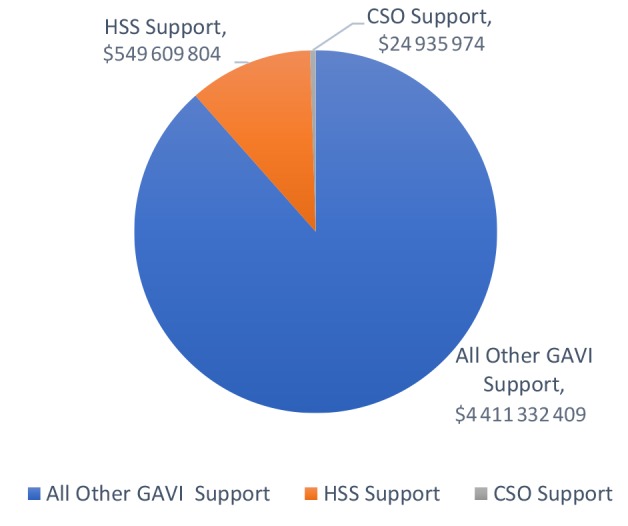
Pattern of GAVI Financial Support to 16 Conflict-Affected Countries.7 Abbreviations: CSO, CSOs, civil society organizations; HSS, health system strengthening.

Between 2010 and 2016, there have been 63 new or underutilised vaccine introductions to the 16 countries under review. Figure 5 illustrates the reported disbursements of funds by GAVI since its inception to the 16 countries.

The civil society investment represents less than 1% of the GHI investment in these countries, and the HSS/CSO investment together represents less than 10% of the overall investment portfolio. GAVI requests that governments finance CSOs through the HSS funding window of support. However, the overall investment in both HSS and CSO windows is less than 12% of the value of the total GAVI investment in the 16 countries under review.

GAVI has implemented several policy options to improve vaccine access for children in these settings. A fragile states policy has been developed, which prioritises ‘country tailored’ approaches to coverage improvement.^77^ Aspects of the fragile states approach by GAVI include support for development of vaccine stockpiles for humanitarian emergencies, reprogramming of HSS support in emerging conflict situations, and finally, increased access by civil society organisations to lower price vaccines in humanitarian emergency settings.^78^ The new vaccines policy also provides a coverage cut off point for new vaccine introduction of 70% DPT3. In support of its overall global goals, GAVI has also developed an increased policy focus on immunisation equity.^79^

## Discussion

### Summary of Main Findings

We found that low immunisation coverage and VPD outbreaks are of major concern in conflict-affected countries, especially in the sub regions most affected by conflict. Common features of the impact of conflict included destroyed infrastructure, depleted human resources, delayed vaccine introductions and delayed campaign implementation, and limited accessibility to services. Outbreaks of VPD have had a substantial impact on attainment of global immunisation disease elimination and eradication goals. This has been confirmed by other studies. The Global Polio Eradication Initiative (GPEI) mid-term strategic plan evaluation demonstrates that conflict and insecurity in the Horn of Africa and the Middle East, and increased instability in Pakistan, are a major factor in limiting access of immunisation services to children.^80^

Lessons from Nigeria, Somalia, and Pakistan illustrate that operational tactics such as security assessments, negotiating secure physical access, engaging local communities, coordination of humanitarian aid deliveries, transit or cross border vaccination strategy, collaboration with the military or other security personnel are all tactical strategies applied to assist reduction of VPD morbidity and mortality in conflict settings.^9^ However, this review has found that many national governments are failing to articulate strategies that meet the needs of displaced or conflict-affected populations within their borders. The limited analysis in multiyear planning documents, HSS strategies and major donor APRs about immunisation service access and conflict raises the question as to why national documentation is so light in this area, when equity of access is clearly a priority for both national governments and Global Health Initiatives such as GAVI.

This may be partly attributable to the fact that in many of these settings, governments are either in conflict with sections of their own populations, or alternately, are not willing to establish health infrastructure and services for populations that have crossed national borders. The stated aims of the Global Health Initiative to expand vaccine access for the most disadvantaged may be problematic when the State is in direct conflict with populations within its own borders. This was evident in a study of the polio outbreak in Syria in 2013 that found that international organisations, due to their mandate to respect the sovereignty of the Syrian government, were not able to prevent and contain disease outbreaks in rebel-controlled areas.^81^

The concentration of poor immunisation coverage and disease outbreaks in conflict-displaced populations reinforces the notion that there is an intertwining of political and health security agendas. This aligns with a recent global report on the ‘weaponization of healthcare’ that recommended improved operational support for health workers working in rebel-controlled areas, including tighter enforcement of principles of medical neutrality in all conflict settings.^82^ This means that there is a need for national planners to seek out solutions through both health and political dialogue. At the local level, Nigeria, Pakistan, and Afghanistan report that the key ingredient for success in conflict settings will most likely result from careful development of a local area communication strategy with local authorities, religious leaders, and combatants.^83^

### Implications for Global Health Policy and Practice

The lack of policy and planning focus on the association between conflict and low immunisation coverage is contributing to wide within-country disparities in immunisation coverage, and, as illustrated by the findings in this paper on polio outbreaks, threatens to derail policy and planning initiatives for global equity in immunisation access. Movement of populations and pathogens across borders demonstrates that cross border approaches are required, as illustrated by either polio or measles disease outbreaks in Central Africa, South Asia, the Middle East, and more recently in Eastern Europe.

Despite the emphasis of Global Health Initiative investments on fragile states and inequity reductions, there is a still a disconnect between the pattern of investment (reflected by high levels of investment in commodities see Figure 5) and the real needs of systems in conflict and post conflict areas (reflected in comparatively lower levels of investment in health systems support). More balanced humanitarian and development assistance portfolios are required in conflict settings, particularly given the stated need of countries for health system support and civil society partnerships. This is attributable in large part to the acute shortage of professional human resources in these areas, and the heavy reliance on NGOs and volunteers to address the gap in service provision. Limitations in health outreach, interruptions to campaigns, and delayed new vaccine introductions all track back to a human resource problem and under investment in the resourcing of health operations.

This recommendation highlights the valuable distinction between health system support and HSS,^84^ with HSS referring to more upstream development of management systems, with the concept of health system support focusing more on provision of essential system inputs for rebuilding routine immunisation services in emergency settings. Critical areas for investment include human resources placement and retention, financing the operational costs associated with ensuring mobility and security for these workers, maintaining the cold chain, and investment in partnerships with civil organisations who have more ready access to hard to reach populations.

## Conclusion

Improved articulation of conflict immunisation strategy in national level immunisation planning and reporting documents will provide significant advantages to managers and populations from several perspectives. Better understanding of operational approaches in conflict settings may enable improved guidance for countries, development partners and for civil agencies in ensuring health worker and community security, as well as improved accessibility to vaccines and related child health interventions for populations in conflict settings. Global policy and strategy dialogue are urgently needed to devise best practice approaches and investment pathways for rebuilding human resources and delivery systems in conflict areas. The widespread impact of conflict on immunisation access and resulting disease outbreaks in Africa, the Middle East, South Asia and more recently in Eastern Europe, provides a strong rationale for improve technical guidance on how planners can mitigate the impact of conflict on the attainment of global health goals.

## Acknowledgements

The authors would like to thank Christalla Hajisava from the University of Melbourne, Melbourne, VIC, Australia for editing and preparing the final submission for publication.

## Ethical issues

Ethical clearance was not sought for this study. Data is from publicly available sources, and no interviews were conducted to undertake this study.

## Competing interests

Authors declare that they have no competing interests.

## Authors’ contributions

JG conducted the research and literature review, and drafted the original version of the paper. BAB reviewed and revised drafts, and provided technical advice on the content and directions of the paper.

## Authors’ affiliations

^1^College of Public Health, Medical and Veterinary Services, Cairns Campus, James Cook University, Douglas, QLD, Australia. ^2^Department of Medicine, Doherty Institute, University of Melbourne, Melbourne, VIC, Australia. ^3^Victorian Infectious Diseases Service, Royal Melbourne Hospital, Parkville, VIC, Australia.

## Key messages

**Implications for policy makers**

The concentration of poor immunisation coverage and vaccine-preventable disease (VPD) outbreaks in conflict-affected populations reinforces the notion that there is *an intertwining of the political and health security agendas*. Inevitably, this means health planners and policy-makers need to seek solutions through both health and political dialogue.

- Global, regional, national and sub-national approaches are critical to addressing imbalances in immunisation service access. Movement of both populations and pathogens across borders demonstrates that cross border approaches are required, as illustrated by VPD outbreaks in Central Africa, South Asia, the Middle East, and more recently in Eastern Europe.
- Humanitarian and development assistance portfolios that reflect a more balanced investment in both commodity and operational support are required in conflict settings, particularly for health system support and civil society partnerships.
- Improved articulation of conflict and post conflict immunisation strategy in national immunisation planning and reporting documents will enable improved guidance and resource allocation for countries, development partners and for civil agencies.
- Increased policy emphasis on immunisation access in conflict-affected areas will assist health worker and community security, as well as improved accessibility to vaccines and related child health interventions.

**Implications for public**

Increased awareness of the impact of conflict on immunisation access should provide a focus for resource mobilisation to ensure health services reach children in these settings. Deeper understanding of the impact of conflict on immunisation coverage will reduce the risk of disease outbreaks in vulnerable populations in conflict affected areas and in internally displaced population camps. This research reinforces a rights approach for children’s health and health workers in conflict settings, and in doing so, has the potential to improve strategy, partnerships and advocacy efforts for both child protection and health worker security.

## References

[1] United States Agency for International Development (USAID). 2017 Demographic and Health Surveys. Demographic Health Surveys website. https://www.dhsprogram.com. Accessed January 24, 2017.

[2] Hussain SF, Boyle P, Patel P, Sullivan R (2016). Eradicating polio in Pakistan: an analysis of the challenges and solutions to this security and health issue. Global Health.

[3] Mashal T, Nakamura K, Kizuki M (2007). Impact of conflict on infant immunisation coverage in Afghanistan: a countrywide study 2000–2003. Int J Health Geographics.

[4] Bagcchi S (2015). Inadequate vaccine coverage fuels polio outbreak in Ukraine. Lancet Infect Dis.

[5] World Health Organization (WHO). 2017 Vaccine Preventable Disease Data base; WHO/United Nations Children’s Fund (UNICEF) Estimates. WHO website. http://www.who.int/immunisation/monitoring_surveillance/data/en/. Accessed January 24, 2017.

[6] World Health Organization (WHO). Disease Incidence Data, 2017. WHO website. http://apps.who.int/immunisation_monitoring/globalsummary/timeseries/tsincidencediphtheria.html. Accessed January 24, 2017.

[7] Global Alliance for Vaccine and Immunisation (GAVI). 2017 Country Hub. Global Alliance for Vaccine and Immunisation website. https://www.gavi.org/country/. Accessed January 24, 2017.

[8] National Institutes for Health. PubMed database. https://www.ncbi.nlm.nih.gov/pubmed. Accessed September 9, 2018.

[9] Nnadi C, Etsano A, Uba B (2017). Approaches to vaccination among populations in areas of conflict. J Infect Dis.

[10] Shuaibu FM, Birukila G, Usman S (2016). Mass immunization with inactivated polio vaccine in conflict zones--Experience from Borno and Yobe States, North-Eastern Nigeria. J Public Health Policy.

[11] Senessie C, Gage GN, von Elm E (2007). Delays in childhood immunization in a conflict area: a study from Sierra Leone during civil war. Confl Health.

[12] Peyraud N, Quéré M, Duc G1 (2018). A post-conflict vaccination campaign, Central African Republic. Bull World Health Organ.

[13] de Lima Pereira A, Southgate R, Ahmed H, O’Connor P, Cramond V, Lenglet A (2018). Infectious disease risk and vaccination in northern Syria after 5 years of civil war: the MSF experience. PLoS Curr.

[14] Silwal RC, Jimba M, Poudyal AK, Poudel KC, Wakai S (2006). Improving immunization services under the armed conflict in rural Nepal. Public Health.

[15] United Nations High Commissioner for Refugees (UNHCR). 2017 Population Statistics. UNHCR website. http://popstats.unhcr.org/en/overview. Accessed January 24, 2017.

[16] Data Sourced from: DHS Statcompiler. STATcompiler website. https://www.statcompiler.com/en/. Accessed January 24, 2017.

[17] Medecines San Frontieres (MSF) Democratic Republic of Congo (DRC): Ongoing conflict in Equateur province Democratic Republic of Congo. Medecines San Frontieres – Doctors Without Borders website. https://www.msf.org.za/stories-news/stories-and-news/drc-ongoing-conflict-equateur-province. Accessed January 24, 2017.

[18] Demographic and Health Survey Pakistan 2012-2013. https://dhsprogram.com/pubs/pdf/FR290/FR290.pdf. Accessed September 15, 2018.

[19] British Broadcasting Corporation (BBC) News. Africa. BBC News website. http://www.bbc.com/news/world-africa-37759749. Accessed January 24, 2017.

[20] Conflict in Yemen: Simple People, Complicated Circumstances Lucas Winter. Middle East Policy Council website. http://www.mepc.org/journal/conflict-yemen-simple-people-complicated-circumstances. Accessed January 24, 2017.

[21] World Health Organisation (WHO). Country Profile Ukraine. http://www.who.int/immunisation/monitoring_surveillance/data/ukr.pdf. Accessed September 8, 2018.

[22] World Health Organisation (WHO). Country Profile Yemen. http://www.who.int/immunisation/monitoring_surveillance/data/yem.pdf. Accessed September 8, 2018.

[23] World Health Organisation (WHO). Country Profile South Sudan. http://www.who.int/immunisation/monitoring_surveillance/data/ssd.pdf. Accessed September 8, 2018.

[24] World Bank. 2017 Population Data. World Bank website. http://data.worldbank.org/indicator/SP.POP.TOTL. Accessed January 24, 2017.

[25] WHO UNICEF. Afghanistan Polio Update October to December 2016. http://applications.emro.who.int/dsaf/AFG/2016/Afg_Polio_oct_dec_2016_19353.pdf?ua=1. Accessed January 24, 2017.

[26] Polio Global Eradication Initiative Afghanistan. http://polioeradication.org/where-we-work/afghanistan/. Accessed September 8, 2018.

[27] Gouandjika-Vasilache I, Mazitchi A, Gumede N (2013). Wild Poliovirus Importation, Central African Republic. Emerg Infect Dis.

[28] World Health Organization (WHO). Poliomyelitis in Chad. WHO website. http://www.who.int/csr/don/2011_06_10a/en/. Accessed January 24, 2017.

[29] World Health Organisation. Poliomyelitis in Chad http://www.who.int/csr/don/2011_06_10a/en/. Accessed September 8, 2018.

[30] Government of Cote D’Ivoire. Multi Year Plan for Immunisation 2007–2011 (update 2013).

[31] UNICEF. Civil Rest has been an Ally in Emergence of Polio. UNICEF website. https://www.unicef.org/infobycountry/cotedivoire_25292.html.

[32] World Health Organisation (WHO). Wild poliovirus in Côte d’Ivoire. http://www.who.int/csr/don/2011_04_21a/en/. Accessed September 8, 2018.

[33] United Nations Office for the Coordination of Humanitarian Affairs (OCHA) DRC. The untold success story of polio. United Nations Office for the Coordination of Humanitarian Affairs website. http://www.unocha.org/top-stories/all-stories/drc-untold-success-story-polio. Accessed January 21, 2017.

[34] World Health Organisation. Circulating vaccine-derived poliovirus type 2 – Democratic Republic of the Congo. http://www.who.int/csr/don/13-June-2017-polio-drc/en/. Accessed September 8, 2018.

[35] World Health Organization (WHO). Ethiopia Country Programmes – Polio. World Health Organization website. http://www.afro.who.int/health-topics/health-topics-ethiopia. Published 2016.

[36] World Health Organization (WHO). POLIO Eradication in ETHIOPIA Progress in 2014. https://www.afro.who.int/sites/default/files/2017-05/ethiopia_update-sheet-on-polio-eradication-programme_2014_final.pdf. Accessed September 8, 2018.

[37] World Health Organization (WHO). Ethiopia launches the 1st round synchronized mOPV2 campaign. https://www.afro.who.int/news/ethiopia-launches-1st-round-synchronized-mopv2-campaign. Accessed September 8, 2018.

[38] WHO: 2013 Wild Polio Virus in the Horn of Africa. World Health Organization website. http://www.who.int/csr/don/2013_05_22/en/. Accessed January 21, 2017.

[39] UNICEF. Myanmar Media Release. https://www.unicef.org/myanmar/media_24943.html. Accessed January 21, 2017.

[40] END Polio. Polio in Pakistan. END Polio Pakistan website. http://www.endpolio.com.pk/polioin-pakistan. Accessed January 21, 2017.

[41] World Health Organization (WHO). Summary of poliovirus circulation in 2016 – Pakistan. http://www.who.int/csr/don/27-december-2016-polio-pakistan/en/. Accessed September 18, 2018.

[42] Polio Eradication Initiative. http://polioeradication.org/wp-content/uploads/2016/09/20160906_AppealNigeria.pdf. Accessed September 4, 2016.

[43] World Health Organization (WHO). Wild polio and vaccine derived polio in Nigeria. http://www.who.int/csr/don/06-october-2016-polio-nigeria/en/. Accessed September 8, 2018.

[44] United Nations Children’s Fund (UNICEF). Polio outbreak in Somalia continues to spread. UNICEF website. https://www.unicef.org/infobycountry/somalia_69874.html. Accessed September 4, 2016.

[45] World Health Organization (WHO). Circulating vaccine-derived poliovirus type 2 – Somalia. http://www.who.int/csr/don/09-March-2018-polio-Somalia/en/. Accessed September 8, 2018.

[46] World Health Organization (WHO). Poliovirus in South Sudan and Madagascar. Disease Outbreak News, November 14, 2014. WHO website. http://www.who.int/csr/don/14-november-2014-polio/en/. Accessed September 4, 2016.

[47] World Health Organization (WHO). Polio Eradication Initiative Sudan. WHO website. http://www.emro.who.int/polio/countries/sudan.html. Accessed September 4, 2016.

[48] World Health Organization (WHO). Polio in Sudan – high risk of international spread. WHO website. http://www.who.int/csr/don/2009_03_02a/en/. Accessed September 9, 2018.

[49] UNICEF. Two million Ugandan children targeted for polio immunisations. UNICEF website. https://www.unicef.org/uganda/media_7547.html. Accessed November 18, 2018. Published 2010.

[50] World Health Organization (WHO). Circulating vaccine-derived poliovirus – Ukraine. WHO website. http://www.who.int/csr/don/01-september-2015-polio/en/. Accessed September 8, 2018.

[51] World Health Organization (WHO). Health Situation in Yemen and WHO response. WHO website. http://www.who.int/hac/crises/yem/yemen-infographic2.pdf?ua=1. Accessed September 8, 2018.

[52] Akil L, Ahmad HA (2016). The recent outbreaks and reemergence of poliovirus in war and conflict-affected areas. Int J Infect Dis.

[53] Government of North Sudan. National Ministry of Health Multi Year Plan for Immunisation 2011-2015.

[54] GAVI. Government of South Sudan Health System Strengthening Strategy 2013. Global Alliance for Vaccine and Immunisation website. http://www.gavi.org/country/south-sudan/. Accessed September 4, 2016.

[55] GAVI. Government of Afghanistan Health System Strengthening Strategy 2015. Global Alliance for Vaccine and Immunisation website. http://www.gavi.org/country/afghanistan/. Accessed September 4, 2016.

[56] GAVI. Government of Pakistan Health System Strengthening Strategy 2016. Global Alliance for Vaccine and Immunisation website. http://www.gavi.org/country/pakistan/. Accessed September 4, 2016.

[57] GAVI. Government of South Sudan Health System Strengthening Strategy 2014. Global Alliance for Vaccine and Immunisation website. http://www.gavi.org/country/south-sudan/. Accessed September 4, 2016.

[58] Government of South Sudan. Annual Progress Report to GAVI 2014. Global Alliance for Vaccines and Immunisation website. https://www.gavi.org/country/south-sudan/documents/. Accessed December 17, 2018.

[59] GAVI. Government of Afghanistan Multi Year Plan for Immunisation 2011-2015. Global Alliance for Vaccine and Immunisation website. http://www.gavi.org/country/afghanistan/. Accessed September 4, 2016.

[60] GAVI. Government of Yemen Annual Progress Report to GAVI 2014. Global Alliance for Vaccine and Immunisation website. http://www.gavi.org/country/yemen/. Accessed September 4, 2016.

[61] GAVI. Government of Kenya Annual Progress Report to GAVI 2014. Global Alliance for Vaccine and Immunisation website. http://www.gavi.org/country/kenya/. Accessed September 4, 2016.

[62] GAVI. Government of Union of Myanmar Multi Year Plan for Immunisation 2007-2011. Global Alliance for Vaccine and Immunisation website. http://www.gavi.org/country/myanmar/. Accessed September 4, 2016.

[63] GAVI. Nigeria Multi Year Plan for Immunisation 2006-2010 (updated 2013). Global Alliance for Vaccine and Immunisation website. http://www.gavi.org/country/nigeria/. Accessed September 4, 2016.

[64] GAVI. Independent Review Committee (IRC) Committee Report New Proposals June-July 2014. Global Alliance for Vaccine and Immunisation website. http://www.gavi.org/support/process/apply/independent-review-committee/. Accessed September 4, 2016.

[65] United Nations Children’s Fund (UNICEF): Côte d’Ivoire. Civil unrest has been an ally in the rise of polio. UNICEF website. https://www.unicef.org/infobycountry/cotedivoire_25292.html. Accessed September 4, 2016.

[66] Nighat JN (2016). The Global Polio Eradication Initiative (GPEI) in Pakistan. JPMA.

[67] HealthMap. At Least Nine Polio Workers Killed in Nigeria. HealthMap Organization website. http://www.healthmap.org/site/diseasedaily/article/least-nine-polio-workers-killed-nigeria-21113. Published February 13, 2013.

[68] GAVI. Somalia Health System Strengthening Strategy. Global Alliance for Vaccine and Immunisation website. http://www.gavi.org/country/somalia/. Accessed September 4, 2016.

[69] GAVI. Government of North Sudan Health System Strengthening Strategy. Global Alliance for Vaccine and Immunisation website. http://www.gavi.org/country/sudan/. Accessed September 4, 2016.

[70] GAVI. Government of the Union of Myanmar Health System Strengthening Strategy 2008. Global Alliance for Vaccine and Immunisation website. http://www.gavi.org/country/myanmar/. Accessed September 4, 2016.

[71] World Health Organisation (WHO). Ukraine conflict: upholding the right to health for all. http://www.who.int/features/2014/ukraine-conflict/en/. Accessed September 8, 2018.

[72] Republic of Central Africa Ministry of Health. Multi Year Plan for Immunisation 2011 – 2015. Global Alliance for Vaccine and Immunisation website. http://www.gavi.org/country/central-african-republic/documents/. Accessed December 8, 2017.

[73] Government of Afghanistan. Health System Strengthening Strategy 2015. https://www.gavi.org/country/afghanistan/documents/.Accessed September 8, 2018.

[74] Government of Yemen. Health System Strengthening Strategy 2014. https://www.gavi.org/country/yemen/documents/. Accessed September 8, 2018.

[75] UNICEF. World Food Program Rapid Response mechanism South Sudan. https://www.unicef.org/appeals/files/WFP_UNICEF_RRM_One_Year_Report.pdf. Accessed September 9, 2018.

[76] GAVI. Democratic Republic of Congo DRC Annual Progress Report to GAVI 2014. Global Alliance for Vaccine and Immunisation website. http://www.gavi.org/country/drc/. Accessed September 4, 2016.

[77] GAVI. Policy on Fragility and Immunisation. Global Alliance for Vaccine and Immunisation website. http://www.gavi.org/about/governance/programme-policies/gavi-policy-on-fragility-and-immunisation/. Accessed September 4, 2016.

[78] GAVI. On the frontline: Gavi’s support to fragile states. https://www.gavi.org/library/news/gavi-features/2016/on-the-frontline--gavi-s-support-to-fragile-states/. Accessed September 9, 2018.

[79] GAVI. Health Equity. Global Alliance for Vaccine and Immunisation website. https://www.gavi.org/about/value/health-equity/. Accessed December 17, 2016.

[80] WHO, Centers for Disease Control (CDC), UNICEF and Gates Foundation. Polio Eradication & Endgame Midterm Review 2015. http://polioeradication.org/wp-content/uploads/2016/07/GPEI-MTR_July2015.pdf. Accessed December 17, 2018.

[81] Kennedy J, Michailidou D (2017). Civil War, Contested Sovereignty, and the Limits of Global Health Partnerships: A case Study of the Syrian polio outbreak in 2013. Health Policy Plan.

[82] The Lancet. The Weaponization of Health Care: Using people’s need for health care as a weapon of war over 6 years of Syrian Conflict. EurekAlert The Global Source of Science News website. https://eurekalert.org/pub_releases/2017-03/tl-tlt031317.php. Accessed September 14, 2017. Published March 14, 2017.

[83] Abimbola S, Malik AU, Mansoor GF (2013). The Final Push for Polio Eradication: Addressing the Challenge of Violence in Afghanistan, Pakistan, and Nigeria. PLoS Med.

[84] Chee G, Pielemeier N, Lion A, Connor C (2013). Why differentiating between health system support and health system strengthening is needed. Int J Health Plann Manage.

